# Supplementary material for: Therapeutic Implications of TGF-β Pathway in Desmoid Tumor Based on Comprehensive Molecular Profiling and Clinicopathological Properties
Source: Cancers (Basel). 2022 Dec 2;14(23):5975. doi: 10.3390/cancers14235975 (PMC9737545; doi:10.3390/cancers14235975)
Supplement: Supplementary file 1 [file cancers-14-05975-s001.zip › SuppleFigure_1109_2.pdf]

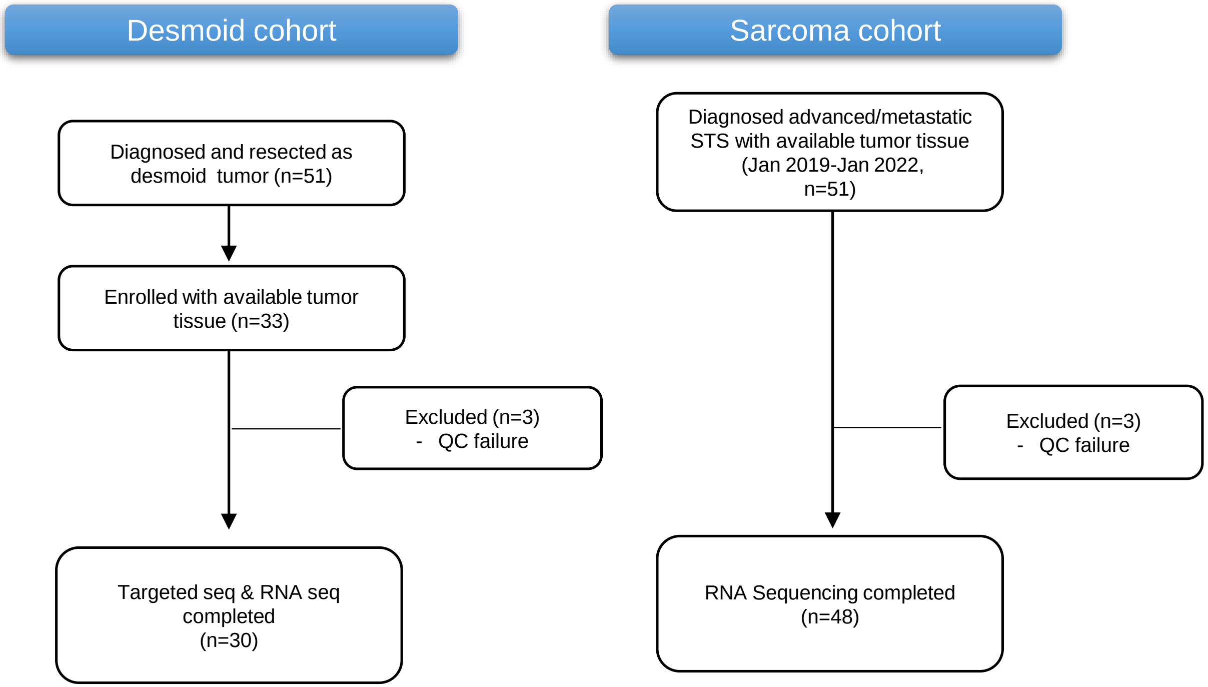

Figure S1. CONSORT diagram

(A)

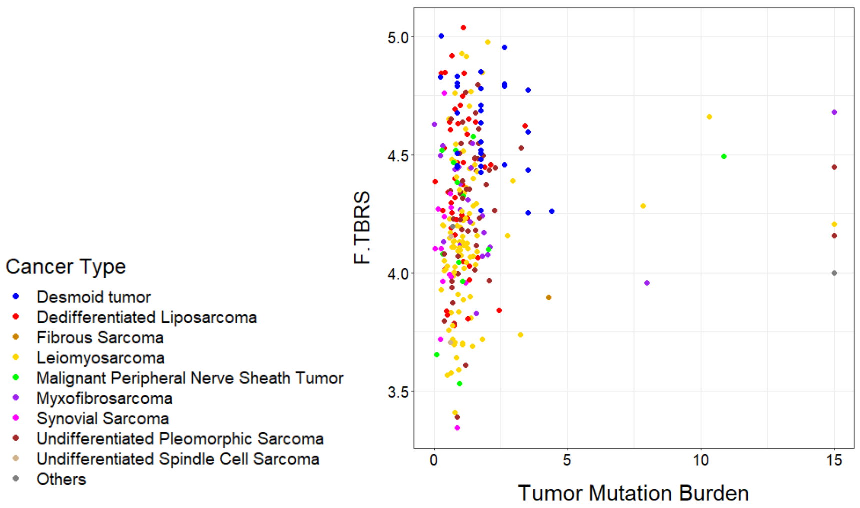

(B)

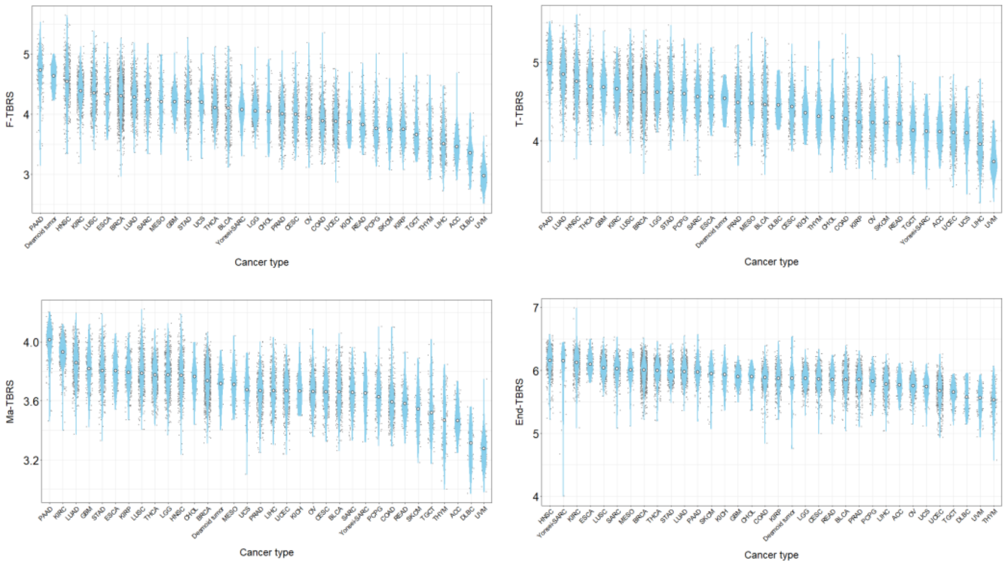

(C)

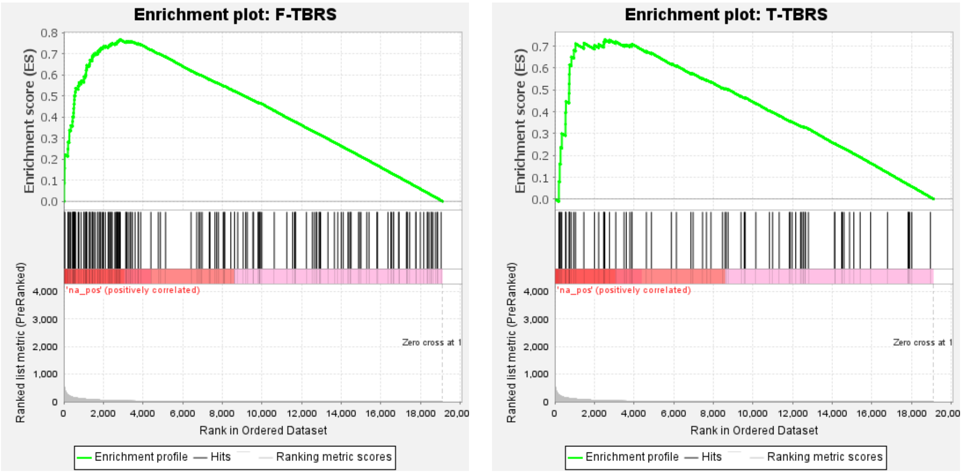

Figure S2. genomic analysis. (A) Boxplots showing TMBs according to cancer type (B) Violin plots showing TBRS scores according to cancer types including TCGA data. Yonsei-SARC indicates sarcoma other than desmoid tumor from this study. Abbreviations: ACC, adrenocortical carcinoma; BLCA, bladder urothelial carcinoma; BRCA, breast invasive carcinoma; CESC, cervical squamous cell carcinoma; CHOL, cholangiocarcinoma; COAD, colon adenocarcinoma; DLBC, diffuse large B cell lymphoma; ESCA, esophageal carcinoma; GBM, glioblastoma multiforme; HNSC, head and neck squamous cell carcinoma; KICH, kidney chromophobe carcinoma; KIRC, Kidney renal clear cell carcinoma; KIRP, kidney renal papillary cell carcinoma; LGG, low grade glioma; LIHC, liver hepatocellular carcinoma; LUAD, lung adenocarcinoma; LUSC, lung squamous cell carcinoma; MESO, mesothelioma; OV, ovarian serous cystadenocarcinoma; PAAD, pancreatic adenocarcinoma; PCPG, pheochromocytoma and paraganglioma; PRAD, prostate adenocarcinoma; READ, rectal adenocarcinoma; SARC, sarcoma; SKCM, skin cutaneous melanoma; STAD, stomach adenocarcinoma; TGCT, testicular germ cell tumor; THCA, thyroid carcinoma; THYM, thymoma; UCS, uterine carcinosarcoma; UCEC, uterine corpus endometrial carcinoma; UVM, uveal melanoma (C)The plots of enrichment score: F-TBRS and T-TBRS.

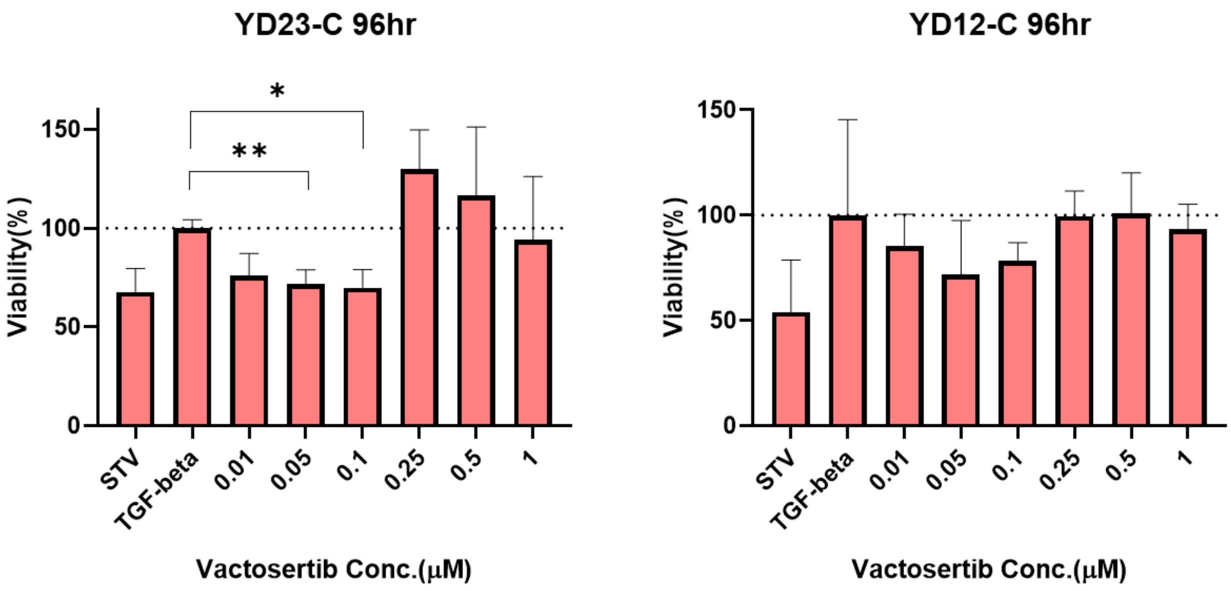

Figure S3. Results of viability test with vactosertib monotherapy in 48hr and 96hr. TGF-beta works as both a tumor suppressor and a tumor promoter. We performed the drug screen for monotherapy of TGF-beta inhibitor (vactosertib) and selected the specific concentration that appeared to have the tumor suppressor effect. And we selected 0.1 μM. Because inhibition of abnormal growth by TGF-beta stimulation in 96hr vactosertib monotherapy was stable in both of PDDs (STV; before TGF-beta stimulation, TGF-beta; TGF-beta 1ng/mL stimulation, all vactosertib treatment was together with TGF-beta).

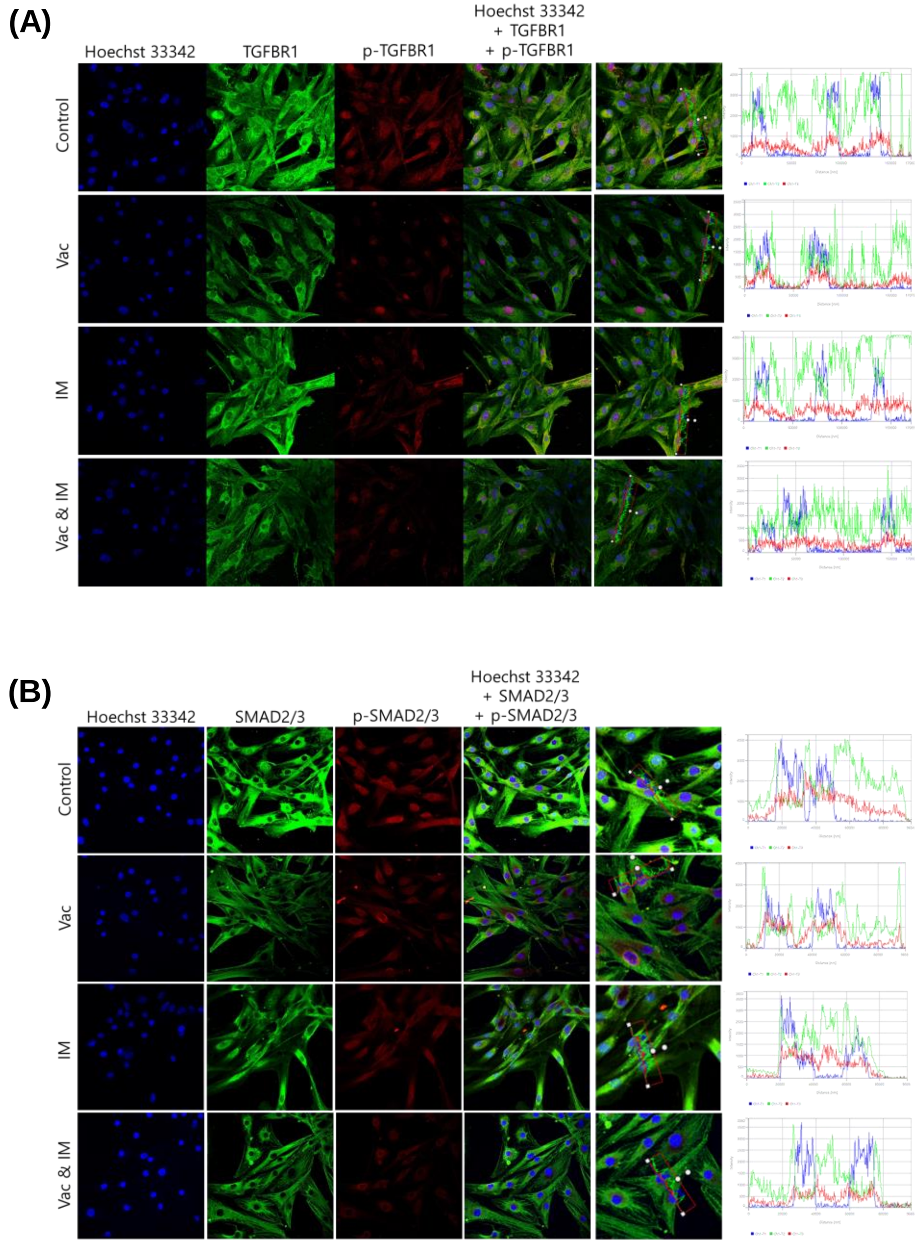

Figure S4. Fluorescence confocal microscopy images and line profiles of YD23-C for TGFBR1 signaling (A) and SMAD2/3 signaling (B).

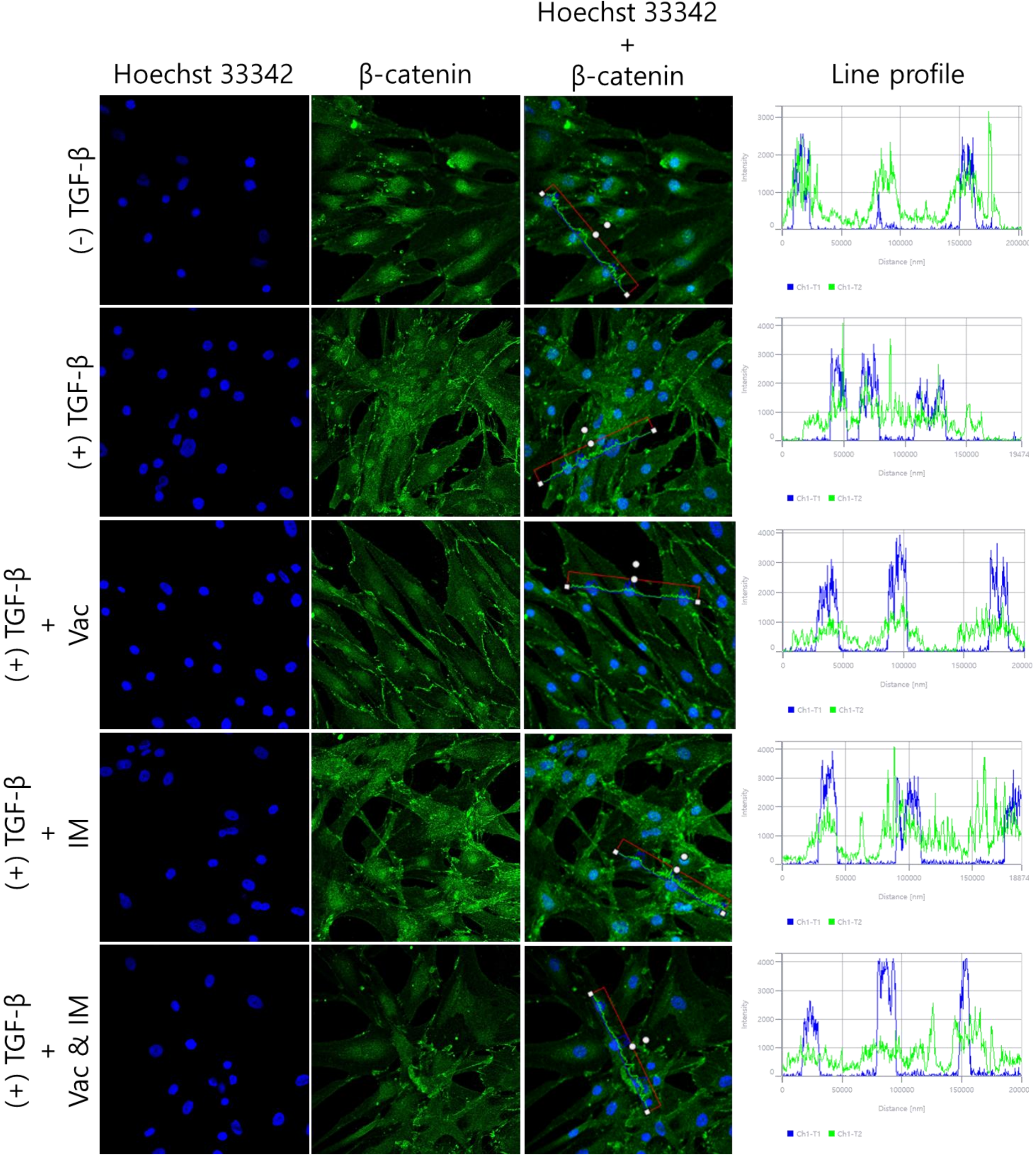

Figure S5. Fluorescence confocal microscopy images and line profiles of YD23-C cells for beta-catenin.
