# Supplementary material for: Therapeutic Implications of TGF-β Pathway in Desmoid Tumor Based on Comprehensive Molecular Profiling and Clinicopathological Properties
Source: Cancers (Basel). 2022 Dec 2;14(23):5975. doi: 10.3390/cancers14235975 (PMC9737545; doi:10.3390/cancers14235975)
Supplement: Supplementary file 1 [file cancers-14-05975-s001.zip › Supplementary table S3.pdf]

Supplementary table.S3 Genertic alteration table about primary tissue and PDC (YD12, YD23).

Hugo _Symbol	NCBI _Build	Chromosome	Start _Position	End _Position	Variant _Type	Reference _Allele	Tumor_Seq _Allele1	Tumor_Seq _Allele2	dbSNP_RS	Tumor_Sample _Barcode	HGVSc	HGVSp	Transcript_ID	Feature	Consequence
FANCD2	hg19	chr3	10106458	10106458	SNP	C	C	T	rs763189891	**YD12-C**	c.2067C>T	p.Tyr689Tyr	NM_033084.4	NM_033084.4	synonymous_variant
CTNNB1	hg19	chr3	41266124	41266124	SNP	A	A	G	rs121913412	**YD12-C**	c.121A>G	p.Thr41Ala	NM_001904.3	NM_001904.3	missense_variant
GNAQ	hg19	chr9	80537095	80537095	SNP	G	G	T	rs200106152	**YD12-C**	c.303C>A	p.Tyr101*	NM_002072.4	NM_002072.4	stop_gained
GNAQ	hg19	chr9	80537112	80537112	SNP	T	T	A	rs753716491	**YD12-C**	c.286A>T	p.Thr96Ser	NM_002072.4	NM_002072.4	missense_variant
FANCD2	hg19	chr3	10106458	10106458	SNP	C	C	T	rs763189891	YD12	c.2067C>T	p.Tyr689Tyr	NM_033084.4	NM_033084.4	synonymous_variant
CTNNB1	hg19	chr3	41266124	41266124	SNP	A	A	G	rs121913412	YD12	c.121A>G	p.Thr41Ala	NM_001904.3	NM_001904.3	missense_variant
GNAQ	hg19	chr9	80537095	80537095	SNP	G	G	T	rs200106152	YD12	c.303C>A	p.Tyr101*	NM_002072.4	NM_002072.4	stop_gained
GNAQ	hg19	chr9	80537112	80537112	SNP	T	T	A	rs753716491	YD12	c.286A>T	p.Thr96Ser	NM_002072.4	NM_002072.4	missense_variant

Hugo _Symbol	NCBI _Build	Chromosome	Start _Position	End _Position	Variant _Type	Reference _Allele	Tumor_Seq _Allele1	Tumor_Seq _Allele2	dbSNP_RS	Tumor_Sample _Barcode	HGVSc	HGVSp	Transcript_ID	Feature	Consequence
RANBP2	hg19	chr2	109371471	109371471	SNP	A	A	G	rs76482726	**YD23-C**	c.2313A>G	p.Ala771Ala	NM_006267.4	NM_006267.4	synonymous_variant
CTNNB1	hg19	chr3	41266137	41266137	SNP	C	C	T	rs121913409	**YD23-C**	c.134C>T	p.Ser45Phe	NM_001904.3	NM_001904.3	missense_variant
RANBP2	hg19	chr2	109371471	109371471	SNP	A	A	G	rs76482726	YD23	c.2313A>G	p.Ala771Ala	NM_006267.4	NM_006267.4	synonymous_variant
CTNNB1	hg19	chr3	41266137	41266137	SNP	C	C	T	rs121913409	YD23	c.134C>T	p.Ser45Phe	NM_001904.3	NM_001904.3	missense_variant
