# Supplementary material for: Therapeutic Implications of TGF-β Pathway in Desmoid Tumor Based on Comprehensive Molecular Profiling and Clinicopathological Properties
Source: Cancers (Basel). 2022 Dec 2;14(23):5975. doi: 10.3390/cancers14235975 (PMC9737545; doi:10.3390/cancers14235975)
Supplement: Supplementary file 1 [file cancers-14-05975-s001.zip › Supplementary Table S4.pdf]

Supplementary Table S4. Results of transcriptomic analyses

**Gene ontology analysis**		
Pathway	p-value	FDR q-value
Hippo signaling pathway	0.00015	0.02189
Pathways in cancer	0.00017	0.02189
Kaposi sarcoma-associated herpesvirus infection	0.00032	0.02731
TGF-beta signaling pathway	0.00045	0.02849
Wnt signaling pathway	0.0009	0.04144
Pathways of neurodegeneration - multiple diseases	0.00098	0.04144
Thermogenesis	0.0014	0.05120
Oxidative phosphorylation	0.0016	0.05120
"Glycosaminoglycan biosynthesis - chondroitin sulfate / dermatan sulfate"	0.002	0.05616
Alzheimer disease	0.0024	0.06163
**Gene set enrichment analysis**		
Pathway	Normalized enrichment score	FDR q-value
EPITHELIAL MESENHCHYMAL TRANSITION	1.35	0.109
WNT BETA CATENIN SIGNALING	1.3	0.081
PANCREAS BETA CELLS	1.3	0.08
ESTROGEN RESPONSE LATE	1.3	0.071
TNFA SIGNALING VIA NFKB	1.3	0.06
HEDGEHOG SIGNALING	1.28	0.064
TGF BETA SIGNALING	1.23	0.158
MYOGENESIS	1.23	0.142
ANGIOGENESIS	1.21	0.155
KRAS SIGNALING UP	1.2	0.172
