# Supplementary material for: Therapeutic Implications of TGF-β Pathway in Desmoid Tumor Based on Comprehensive Molecular Profiling and Clinicopathological Properties
Source: Cancers (Basel). 2022 Dec 2;14(23):5975. doi: 10.3390/cancers14235975 (PMC9737545; doi:10.3390/cancers14235975)
Supplement: Supplementary file 1 [file cancers-14-05975-s001.zip › Supplimentary Table S2..pdf]

Supplimentary Table S2. Patient demographic and clinicopathologic characteristics (Total N=48)

		No.	%
Sex			
	Male	23	47.9
	Female	25	52.1
Age, year			
	Median (range)	57	(32-80)
Histology by Lineage			
	Leiomyosarcoma	13	27.1
	Liposarcoma[†]	8	16.7
	Undifferentiated pleomorphic sarcoma	5	10.4
	Angiosarcoma of soft tissue	5	10.4
	Malignant peripheral nerve sheath tumor	3	6.3
	Synovial sarcoma	2	4.2
	Chondrosarcoma	2	4.2
	Endometrial stromal sarcoma	2	4.2
	Others[‡]	8	16.7
FNCLCC grade			
	1	2	4.2
	2	7	14.6
	3	17	35.4
	Unknown	22	45.8
Primary site			
	Abdomen and thracic visceral organs	21	41.2
	Trunk or retroperitoneum	12	23.5
	Extremity	8	15.7
	Head and neck	7	13.7
Metastatic site			
	Lung	30	62.5
	Bone	15	31.3
	Liver	13	27.1
	Lymph node	5	10.4
	Peritoneum	5	10.4
	Others[¶]	9	18.8
Previous surgery history			
	Yes	37	77.1
	No	11	22.9
Previous radiotherapy history			
	Yes	27	56.3
	No	21	43.8

†Liposarcoma histology include myxoid/round cell liposarcoma (n=4), liposarcoma (n=3), and well-differentiated liposarcoma (n=1).
‡Other histology include alveolar soft part sarcoma; carcinosarcoma; desmoplastic small round cell tumor; embryonal rhabdomyosarcoma; epithelioid hemangioendothelioma; epithelioid sarcoma; intimal sarcoma; solitary fibrous tumor, malignant(each n=1).
¶Other metastastic sites include nasal cavity (n=1); muscle (n=3); pericardium (n=1); brain (n=1); colon (n=1); adrenal gland (n=1); pancreas (n=1).
FCLCC, Fédéra- tion Nationale des Centres de Lutte Contre Le Cancer
