# Supplementary material for: Therapeutic Implications of TGF-β Pathway in Desmoid Tumor Based on Comprehensive Molecular Profiling and Clinicopathological Properties
Source: Cancers (Basel). 2022 Dec 2;14(23):5975. doi: 10.3390/cancers14235975 (PMC9737545; doi:10.3390/cancers14235975)
Supplement: Supplementary file 1 [file cancers-14-05975-s001.zip › Supplmentary Table S1.Desmoid patient.pdf]

Supplimentary Table S1 Patient characteristics of desmoid cohort (Total N=30)

		No.	%
Sex			
	Male	18	60
	Female	12	40
Age, year			
	Median (range)	44	(19-68)
Tumor site			
	Abdominal wall	3	10
	Intra-abdominal	11	36.7
	Extra-abdominal	16	53.3
	Trunk	5	
	Upper Extremities	5	
	Neck	3	
	Lowe extremities	3	
FNCLCC grade			
	1	2	4.2
	2	7	14.6
	3	17	35.4
	Unknown	22	45.8
Previous surgery history			
	No	3	10
	Yes	27	90
Previous chemotherapy history			
	No	23	76.7
	Yes	7	23.3
	Imatinib	6	
	Vinblastine/MTX	2(1)*	
Previous radiotherapy history			
	No	26	86.7
	Yes	4	13.3
Recurrency			
	Yes	16	53.3
	No	14	46.7

*1 patient received the chemotherapy for imatinib as well as vinblastine/MTX
